# Vertical distribution of ambient air pollutants (PM_2.5_, PM_10_, NO_X_, and NO_2_); A systematic review

**DOI:** 10.1016/j.heliyon.2024.e39726

**Published:** 2024-10-24

**Authors:** Vahid Roostaei, Farzaneh Gharibzadeh, Mansour Shamsipour, Sasan Faridi, Mohammad Sadegh Hassanvand

**Affiliations:** a Department of Environmental Health Engineering, School of Public Health, Tehran University of Medical Sciences, Tehran, Iran; b Center for Air Pollution Research (CAPR), Institute for Environmental Research (IER), Tehran University of Medical Sciences, Tehran, Iran; c Department of Research Methodology and Data Analysis, Institute for Environmental Research (IER), Tehran University of Medical Sciences, Tehran, Iran

**Keywords:** Air pollution, Particulate matter, NO_2_, Vertical profile

## Abstract

**Background:**

Numerous investigations have explored variations in vertical air pollutant concentrations, yielding diverse findings. So, we have conducted this systematic review to gain a more comprehensive understanding of the vertical distribution of air pollutant concentrations (PM_2.5_, PM_10_, NO_X_, and NO_2_) and the influencing factors.

**Methods:**

Relevant studies were identified by searching the three central electronic databases, PubMed, Web of Science, and Scopus, from the beginning of 2000 to the end of 2023. This study included original articles published in English that examine the pollutant concentration variations below 500 m.

**Finding:**

Of 3614 articles, 57 studies met our criteria. Our findings showed a decline in PM_2.5_ and NO_X_ concentrations as altitude increased, while NO_2_ concentrations exhibited an increase. Conversely, no statistically significant relationship was identified between altitude and PM_10_ concentrations. The regression analysis yielded coefficients for the relationship between concentration and altitude (0–500 m) as follows: PM_2.5_ (−0.11), PM_2.5_/PM_10_ (0.0008), NO_X_ (−0.11), and NO_2_ (0.13). Conducting additional research on this topic and investigating the impact of meteorological parameters on pollutant concentrations at different altitudes enhances our understanding of the vertical distribution of pollutant concentrations.

## 1 Introduction

Airborne particles are regarded as one of the most significant air pollutants globally, and exposure to fine particulate matter (PM_2.5_) can lead to premature death and various chronic and acute illnesses [1]. The Global Burden of Disease (GBD) study findings have revealed that air pollution is the sixth leading cause of death worldwide, making it the most crucial environmental risk factor with the highest burden of disease [2], such as asthma, autoimmune diseases, cardiovascular diseases, type 2 diabetes, neurological diseases, digestive diseases, and cancer [3]. Long-term exposure to PM_2.5_ caused 3.8 million deaths in 2019 [4]. Prominent mechanisms that underlie the adverse effects of air pollution include oxidative stress, systemic inflammation, endothelial dysfunction, autonomic imbalance, thrombogenicity, increased autonomic nervous system activity, vasoconstriction, rheological changes [[5], [6], [7]].

PM_2.5_ is emitted primarily from natural sources, including soil dust and sea salt, and anthropogenic sources, such as exhaust of motor vehicles, waste burning, and industrial processes. It can also be generated secondarily through physical processes, such as condensation, coagulation, and surface coating reactions, and chemical processes, such as photochemical reactions and gaseous precursors undergo chemical reactions being converted to particles [8,9].

NO_**X**_ is a term used to refer to the sum of nitrogen oxide (NO) and nitrogen dioxide (NO_2_) gases, a significant precursor to acid deposition. The photochemical reaction between ozone (O_3_) and NO_**X**_ is responsible for causing severe photochemical air pollution [10]. Nitrogen dioxide (NO_2_) plays a crucial role in the troposphere as it contributes to the creation of tropospheric ozone and nitrate particles, making it a significant gas that significantly affects the atmosphere's quality [11]. Also, the presence of NO_2_ gas can lead to the formation of acid rain, which can harm the environment and human health. When NO_2_ is an aerosol, it can also decrease horizontal visibility. Furthermore, NO_2_ gas is a crucial contributor to the development of peroxyacetyl nitrate (PAN) [12]. As cities expand and populations and car usage rise, the primary sources of NO_2_ emissions and the exposure of urban residents to this gaseous pollutant increase. The mechanisms that NO_2_ causes adverse effects have not distinguished from those of PM and include the induction of increased levels of oxidative free radicals and inflammation [13].

In recent decades, due to urbanization growth and space limitations [9], living in high-rise buildings has become widespread, and people's living and working spaces have shifted to greater heights from ground level. Studies showed that the mass concentration, size distribution, and chemical composition of air pollutants can vary significantly based on altitude in an urban atmosphere [14]. Primary pollutants typically originate from ground-level sources and can disperse vertically through the atmosphere. During the vertical mixing process, chemical reactions occur, forming secondary pollutants. As a result, individuals residing at varying heights may be exposed to different concentrations of pollutants. Consequently, knowledge of the vertical distribution of pollutants is essential for assessing people's exposure to air pollution [15,16].

The upward movement, dispersion, diffusion, accumulation, and deposition of the pollutants are significantly influenced by the location, source emissions, and meteorological factors such as air temperature, wind speed and wind direction, relative humidity, pressure, precipitation, and also planetary boundary layer (PBL) dynamics affecting the concentration of the pollutants [8,12,14,17]. So varied and conflicting observations have been reported in the studies. In a study by Choomanee and his colleagues in Bangkok, at heights of 30, 70, and 110 m, it was observed that the concentration of PM_2.5_ increases with increasing height both during the day and at night [18]. Another study found that under slightly unstable stratification, the concentration remained relatively constant with height increments for southwesterly and southerly winds. In contrast, in north, west, and northwest winds, an increase in concentration was observed in height. For other wind directions, the concentration decreased as height increased by approximately 40 %–50 % [19]. Xiao et al. demonstrated a decreasing trend in the vertical distribution of PM_2.5_ mass concentrations with increasing height in residential areas [20]. In the other study in which the concentration of PM_2.5_ was monitored from a height of 1.5–89.1 m (from the 1st to the 27th floor), the trend of concentration changes with increasing height varied at different times and different heights [21]. Contradictory findings were also reported for NO_2_; a decreasing trend was observed in some studies following increasing the altitude [9,10,14,[20], [21], [22]]. However, some researchers demonstrated an increasing trend [22]. No significant dependency on height was also reported [23]. Moreover, several different patterns, including decrease with increasing height up to a specific height and then increase, or vice versa, and uniform vertical distribution, were demonstrated in some studies [12,[24], [25], [26]]. The variations in observations can be ascribed to differences in measurement season, time of day and night, each region's unique environmental and meteorological conditions, and location properties.

The apparent contradiction within the reported observations shows the necessity of conducting a systematic review to formulate a broad conclusion. Therefore, this study aims to review relevant literature and consider conducting conditions and measured parameters to conclude concentration variations with increasing altitude comprehensively.

## 2 Methods

### 2.1 Identification of studies and search strategy

The search strategy is a fundamental component of a systematic review, developed to identify and retrieve accurate and relevant results effectively. Relevant literature was identified by searching through three central electronic databases: PubMed, Web of Science, and Scopus from the beginning of 2000 to the end of 2023 to find studies on the vertical distribution of air pollutants (PM_2.5_, PM_10_, and NO_2_).

The search words/terms were selected based on the knowledge of the contributing authors and included two components as follows [1]: (PM_10_) OR ("particulate matter") OR (PM_2.5_) OR ("coarse particle") OR ("fine particle" OR ("fine PM") OR ("ultrafine particle") OR (UFP) OR (NO_2_) OR (NO_**X**_) OR ("nitrogen dioxide") AND [2] ("vertical distribution") OR ("vertical profile") OR ("vertical trend") OR ("vertical variation")

### 2.2 Selection criteria

Since the ultimate purpose of the present study, which aims to review the concentration variations of air pollutants with increasing altitude, is to investigate individuals' exposure, and most of them reside at a maximum altitude of 500 m, so we included only articles that examine concentration variations below this height. Moreover, only original articles published in English were included (excluding letters to editors, review articles, and conference presentations). We also excluded articles that showed the concentration of air pollutants (PM_2.5_, PM_10,_ and NO_2_) at various altitudes in graphs that could not be quantified with reasonable accuracy.

### 2.3 Studies selection

Two authors (V. R. and F. G.) independently screened the titles, abstracts, and full text of identified articles for eligibility. Disagreements were resolved by discussion or consultation with a third author (M. S. H.). Afterward, the full text of the remaining articles was thoroughly reviewed, and those who met the criteria were selected for the study. Any discrepancy was consulted until a consensus was reached.

### 2.4 Data extraction

We primarily extracted data on the height and concentration of pollutants from various studies. Additionally, if the studies provided information about the measurement location, date of measurement, time of measurement, type of measurement site, distance from streets, and meteorological parameters (temperature, relative humidity, wind speed), they were also included.

### 2.5 Statistical analysis

We employed a two-step regression analysis to investigate the impact of altitude, meteorological parameters (temperature, humidity, and wind speed), and proximity to the street on pollutant concentrations. Initially, univariate regression analyses were performed to assess the independent effects of each variable. Subsequently, forward and backward stepwise regression analyses were conducted using different significance thresholds (P-value cut points of 0.1 and 0.2) for variable inclusion and exclusion, as detailed in Table 4. This approach was chosen to prioritize identifying key predictors and exploring underlying relationships rather than to test an extensive set of hypotheses. To ensure the robustness and reliability of our models, we evaluated different regression models using comprehensive indicators such as R^2^, adjusted R^2^, and root mean square error (RMSE). These metrics allowed us to assess the overall fit, predictive accuracy, and model complexity. All statistical analyses were conducted using Stata (version 17) and Excel (2019) software, with a standard significance level (P-value) set at 0.05. This strategy carefully balanced the need to identify meaningful predictors with the risk of overfitting, resulting in a robust and interpretable analysis of the factors influencing pollutant concentrations.

Furthermore, due to variations in geographical locations, different levels of air pollution, and diverse altitudes at which the studies were conducted, making direct comparisons between them can lead to misleading conclusions. Hence, we computed the changes in pollutant concentrations per meter of elevation increase to facilitate a more precise comparison. Additionally, we normalized the secondary concentrations at each altitude by dividing them by the baseline concentration specific to each study. Using the following formula:

$$\frac{\left(\frac{C_{2}-C_{1}}{C_{1}}\right)}{\left(H_{2}-H_{1}\right)}\times 100$$

C_2_ means the concentration of pollutants at any altitude in a study, and C_1_ implies the concentration of pollutants at baseline height in the same study. Then H_2_ means the height of the C_2_. Finally, H_1_ is the baseline height of the study.

## 2 Results

### 2.1 Search result

**Fig. 1:**
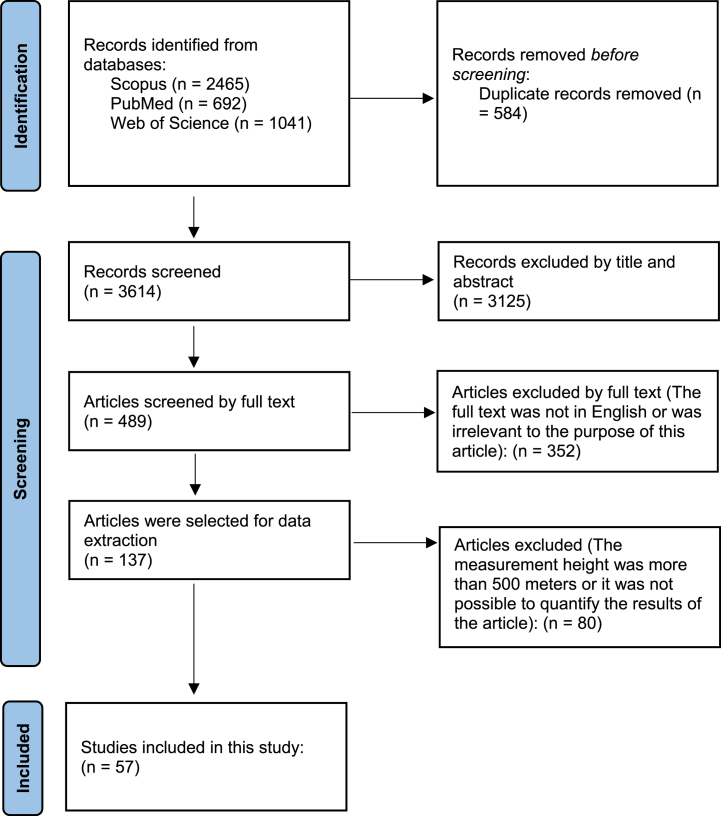
PRISMA flow diagram.

3614 articles were identified using a search strategy and in the database based on the title, abstract, and keywords. After removing duplicates, 584 records were included, and finally, 57 of them met the defined inclusion criteria by reviewing the full texts. The steps of the study selection process are presented in the PRISMA flow diagram (Fig. 1).

### 2.2 Overview of the included studies

**Fig. 2:**
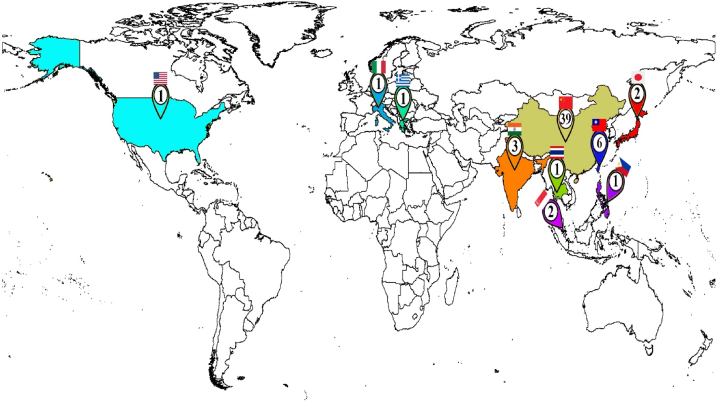
The spatial distribution included in the studies (n = 57) according to publication number.

The spatial distribution of studies by country is illustrated in Fig. 2. Out of the 57 reviewed studies that met our quantitative and qualitative inclusion criteria, 54 studies were conducted in Asia (China, India, Singapore, Thailand, Taiwan, Philippines, Japan), two in Europe (Italy, Greece), and one in Northern America (USA). Information about the country, city, number, and year of studies is provided in Table S1 (in supplementary data). The distribution of the studies by the year of publication is shown in Fig. S1. As observed, the number of studies has increased since 2018 compared to before; the number was 1–4 per year. This trend kept growing until 2021(11 (19.3 %) publications), and the number of studies decreased. Measurement heights in the articles have been presented in Fig. S2 as a function of the date. It can be seen with the advancement of technology that measurement heights have risen in recent years. Among the pollutants considered in this study, it was found that most studies (42(73.7 %)) had focused on investigating the concentration variations of PM_2.5_. Meanwhile, 31.6 %, 26.3 %, and 5.3 % of the 57 studies had examined the concentration variations of PM_10_, NO_2_, and NO_x_, respectively. The majority of the studies were conducted in urban areas (35.08 %), followed by traffic sites (26.3 %), commercial (12.3 %), industrial (12.3 %), residential (7 %), and urban background (3.5 %). Additionally, three individual studies occurred in background, rural, and coastal sites, while the study site was not specified in five studies. Some included studies reported distance from the street and meteorological parameters. Distance from the street had been reported in 24 studies (42.1 %). Also, some of them had measured meteorological parameters, including temperature (9 studies (15.8 %)), relative humidity (8 (14.03 %)), wind speed (6 (10.5 %)), and wind direction (2 (3.5 %)). Detailed information is presented in Table 1.

**Table 1:** Summary of studies.

Study NO. (References)	Country, City	Measurement date	Site of study	Type of pollutant	Measured parameters	Measuring device
1 [27]	Philippines, Quezon City	2018	Urban	PM_2.5_	T∗, WS∗, RH∗	Unmanned Aerial Vehicles (UAV)
2 [28]	China, Shandong	2020	Suburban	PM_2.5,_ PM_10_	DS∗	UAV
3 [18]	China, Beijing	2005	Urban & suburban	PM_2.5,_ PM_10_	–	portable air samplers
4 [29]	Hong Kong	2000	Urban	PM_2.5,_ PM_10_	DS	Portable Air Samplers
5 [25]	Taiwan	2001	Traffic	NO_2_	–	tethered balloon
6 [30]	Taiwan	2020	Urban	PM_2.5,_ PM_10_	–	portable aerosol monitors
7 [31]	Thailand, Bangkok	2020	Urban	PM_2.5_	T, WS, RH	area dust monitor
8 [32]	China, Guangzhou	2014	NR∗	PM_2.5,_ PM_10_	–	a GRIMM 180 aerosol particle spectrometer
9 [15]	China, Beijing	2005	Urban	PM_2.5,_ PM_10_	–	TSI Dust Track Model 8520 Aerosol monitors
10 [33]	India, Chennai	2015	Commercial, traffic	PM_2.5,_ PM_10_	DS	high volume samplers
11 [34]	India, Chennai	2021	Commercial, Residential	PM_2.5,_ PM_10_	DS	Am bient Fine Dust Samplers
12 [16]	China, Shanghai	2017	Traffic	PM_2.5_	DS	TSI Sidepak AM510 detector
13 [22]	China, Jilin	2021	Industrial, Commercial	PM_2.5,_ PM_10,_ NO_2_	–	UAV
14 [35]	China, Xi'an	2018	Urban water, green space, road	PM_2.5_	–	UAV
15 [36]	China, Tianjin	2009	Urban	NO_**X**_	–	nitrogen oxides analyzer (model 200E)
16 [37]	China, Tianjin	2015	Traffic, residential	PM_2.5,_ PM_10_	–	ambient particulate monitor chemiluminescence
17 [38]	Taiwan, Taipei City	2018	Urban	PM_2.5_	–	MET-ONE 1020
18 [39]	China, Luoyang	2021	Urban	NO_2_	WS	MAX-DOAS
19 [40]	China, Lanzhou	2019	Residential, traffic	PM_2.5,_ NO_2_	–	Ogawa badges (NO_2_) DataRAM pDR1000 (PM_2.5_)
20 [41]	Singapore	2008	Traffic	PM_2.5_	DS	Mini Vol samplers
21 [11]	China, Beijing	2021	Urban	NO_2_	DS	MAX-DOAS
22 [42]	Greece, Patras	2020	Traffic	PM_10_	DS	portable sampler (ZAMBELLI MOD.5000)
23 [43]	Japan, Tokyo	2011	Urban, traffic	PM_2.5_	DS	INF sampler
24 [44]	China, Pearl River Delta	2019	Urban	PM_2.5,_ NO_**X**_	T, WS, RH, DS	Thermo scientific™ 5030i & 42i gas analyzer
25 [9]	China, Shanghai	2020	Industrial	PM_2.5_	–	SidePak™ AM510, TSI, USA
26 [26]	China, Shanghai	2018	Coastal	PM_2.5_	–	UAV (TSI AM510)
27 [26]	China, The Yangtze River Delta	2018	Urban background	PM_2.5_	–	UAV (SidePak™, Model: AM510)
28 [45]	China, Shanghai	2007	Traffic	PM_2.5_	DS	DustTrak, TSI Model 8520 Aerosol Monitor
29 [46]	Taiwan, Taipei	2019	Commercial, traffic, industrial	PM_2.5_	DS	multiple Harvard Impactors
30 [47]	Taiwan, Taipei	2021	Commercial, traffic, industrial	PM_2.5_	DS	multiple Harvard Impactors
31 [48]	China, Macau	2019	Urban	PM_2.5_	T, RH	UAV
32 [49]	China, Nanjing	2018	Urban	PM_2.5,_ PM_10_	DS	air sampler, DustTrak, II Model 8532,
33 [50]	China, Shanghai	2021	Traffic	PM_10_	DS	UAV
34 [51]	China, Lin'an	2019	Suburban	PM_2.5_	T, RH	UAV
35 [12]	China, Beijing	2007	Urban	NO_2_	–	Passive sampling
36 [23]	Japan, Tokyo	2014	Traffic	NO_2_	DS	O3 chemiluminescence (Model 42i-TL, Thermo Electron)
37 [52]	India, Delhi city	2021	Urban background	PM_2.5,_ PM_10_	–	high volume sampler, APM 460 NL
38 [53]	Italy, Bologna	2017	Urban	PM_2.5,_ NO_2_	DS	PM_2.5:_ gravimetric samplers (Skypost PM, TCR TECORA) NO_2_: radial symmetry diffusive samplers
39 [54]	USA, Texas	2004	Urban	NO_2_	DS	differential optical absorption spectroscopy (DOAS)
40 [55]	China, Shanghai	2019	Industrial	PM_2.5_	T, RH	UAV
41 [56]	China, Macao	2002	Traffic	PM_2.5,_ PM_10_	DS	The DustTrak Aerosol
42 [57]	China, Guangzhou	2003	Traffic	NO_2_	DS	24-h automatic monitoring system
43 [21]	China, Xi'an	2021	NR	PM_2.5,_ PM_10_	DS	GRIMM1.109
44 [58]	China, Tianjin	2011	Commercial and residential	PM_10_	–	NR
45 [59]	China, Shanghai	2020	Traffic	PM_2.5,_ PM_10_	T, RH	UAV (TSI DustTrak 8534)
46 [60]	China, Guangzhou	2020	Urban	PM_2.5_	–	Online measurements (Model 5030i SHARP, Thermo)
47 [61]	Singapore	2014	Residential and traffic	PM_2.5_	DS	dust monitors
48 [10]	China, Beijing	2006	Traffic	NO_**X**_, NO_2_	DS	42 CTL trace level chemiluminescence NO-NO2-NO_**X**_ analyzers
49 [62]	USA, Boston, Chinatown	2014	Commercial and residential	PM_2.5_	DS	SidePak Aerosol Monitor TSI AM51
50 [63]	China, Lushan Mountain	2019	NR	PM_2.5_	Ds	DustTrak DRX 8533 (TSI, USA)
51 [64]	China, Shanghai	2017	Industrial	PM_2.5_	–	The AM520 p.m.2.5 monitors
52 [65]	China, Wangdu	2018	NR	PM_2.5_	T, WS, WD∗, and water vapor mixing ratio	Mini Air Station-AF300, China, mounted on the tethered balloon
53 [66]	China, Yangtze River Delta	2020–2021	Industrial	PM_2.5_, NO_2_	–	UAV
54 [67]	China, Rao yang	2014	Rural	NO_2_	T, WS, WD, RH	Mini MAX-DOAS system
55 [66]	China, coast of Bohai Sea	2020–2021	NR	NO_2_	–	MAX-DOAS instrument
56 [68]	Taiwan, Taipei metropolis	2020	Urban	PM_2.5_	–	Pall Corporation, Ann Arbor, MI, USA
57 [69]	China, Longfengshan	2020–2021	Background	NO_2_	–	MAX-DOAS

T∗: Temperature.

WS∗: Wind speed.

WD∗: Wind direction.

RH∗: Relative humidity.

DS∗: Distance from street.

NR∗: not reported.

The distribution of the studies by measurement time revealed that most studies (27 (47.3 %)) were done both day and night. In 23 studies (40.3 %), the measurement work was carried out only during the day, and in two studies (3.5 %), it was conducted only at night. The time of the examination was not mentioned in 5 studies (8.7 %).

**Fig. 3:**
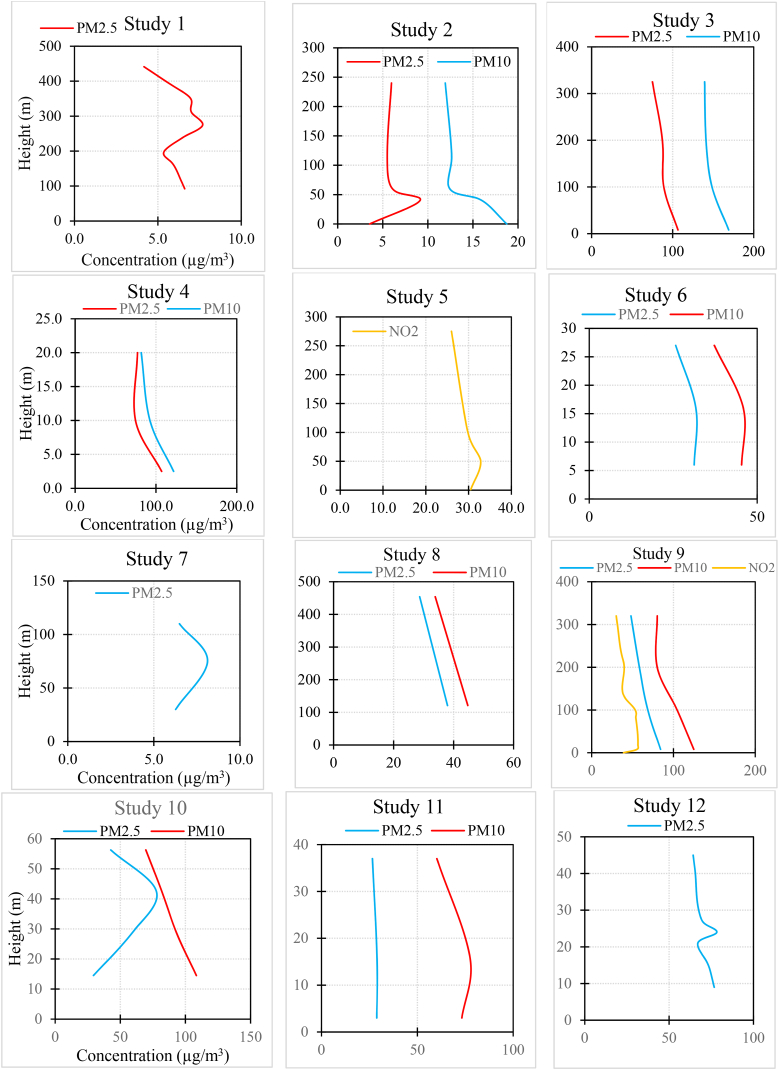

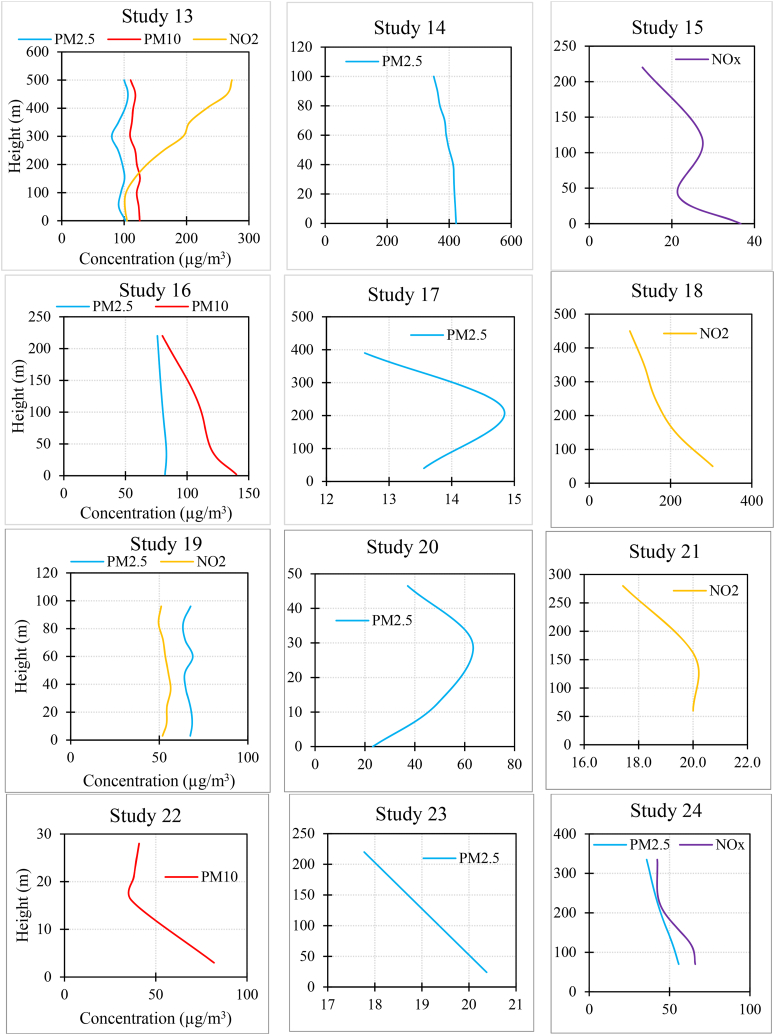

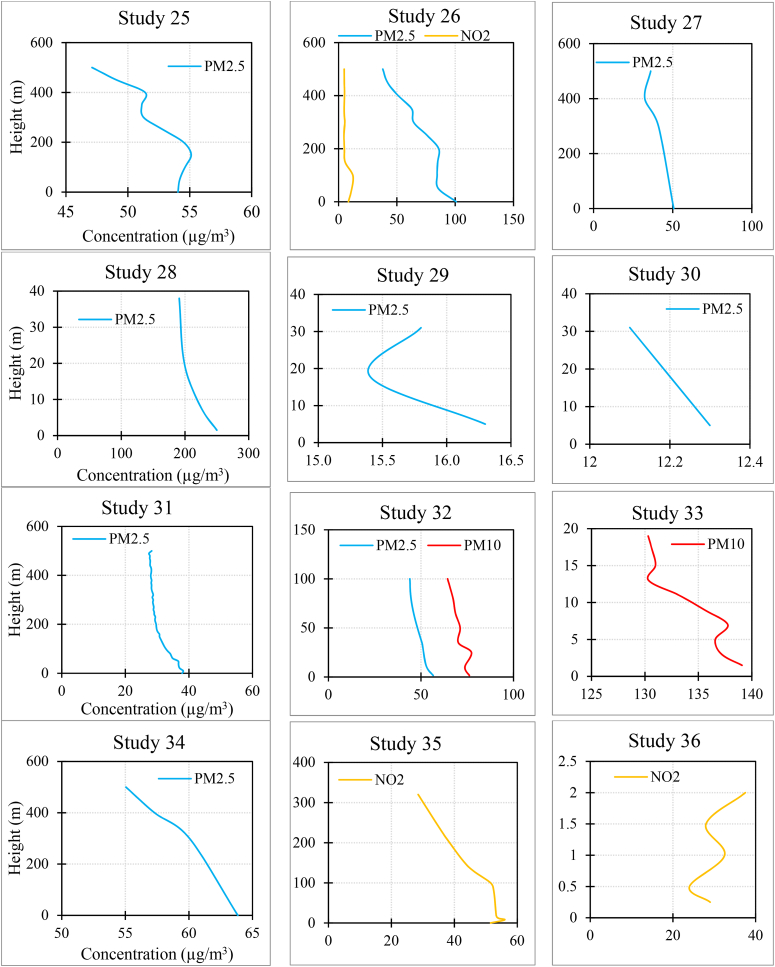

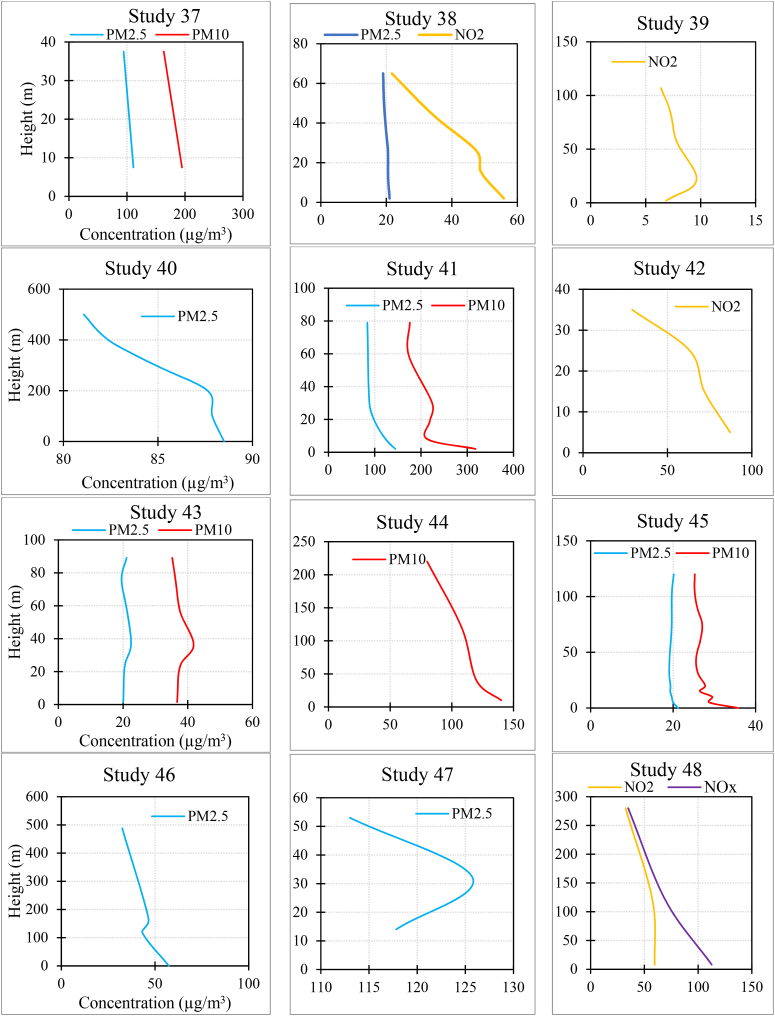

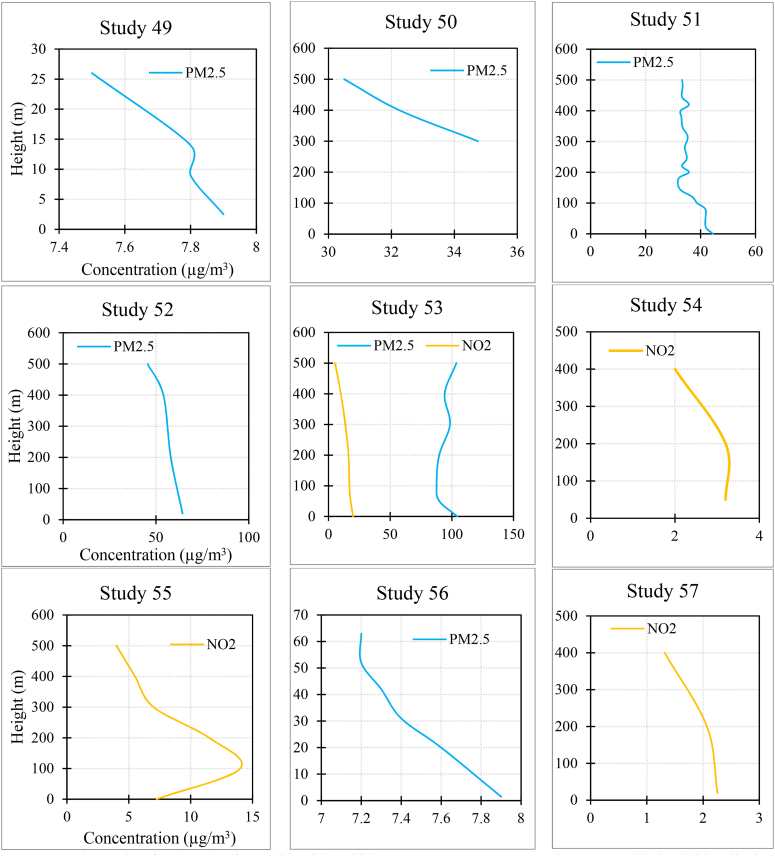
The results of each study (for studies with multiple measurements, average results were provided. Additionally, in studies measuring various pollutants, the measurements might not have been taken simultaneously).

Fig. 3 and Table S2 summarize the results of each study. In the case of PM_2.5_, 15.3 % of studies found that the concentration increased as the height increased. Also, 58.9 % of studies found that it decreased with increasing altitude, and 25.6 % of the studies did not observe a fixed trend (e.g., the concentration first increased and then decreased with height, or one measurement indicated an increase in concentration with height while another measurement showed a decreasing trend).

In the case of PM_10_, 6.6 % of studies found that the concentration increased as the height increased. Also, 60 % of studies found that it decreased with increasing altitude, and 33.3 % of the studies did not observe a fixed trend.

In the case of NO_2_, 18.7 % of studies found that the concentration increased as the height increased. Also, 56.2 % of studies found that it decreased with increasing altitude, and 25 % of the studies did not observe a fixed trend.

In the case of NO_**X**_, all the studies found that the concentration decreased as the height increased.

### 2.3 Air pollutants and height correlation

Table 2 illustrates the regression of air pollutants with height, meteorological parameters, and distance from the street. A statistically significant relationship exists between altitude and air pollutants (PM_2.5_, NO_X_, and NO_2_). With increasing altitude, PM_2.5_ and NO_X_ concentrations decrease while the concentration of NO_2_ increases. Also, no statistically significant relationship was found between PM_10_ concentration and altitude.

**Table 2:** Pollution regression with altitude, meteorological parameters, and distance from street.

PM_2.5_	Simple Reg			Stepwise Reg				MEP#
	Coeff (Cons)	CI (95 %)		Forward stepwise reg (0.20)	Forward stepwise reg (0.10)	Backward stepwise reg (0.20)	Backward stepwise reg (0.10)	Forward stepwise reg (0.1)
Height (0–500m)	−0.11^c^ (94.32)	−0.14	−0.09	–	–	–	–	0.455^a^
Temperature	−2.28^c^ (80.5)	−2.80	−1.76	6.62^a^	6.62^a^	–	–	–
Windspeed	−32.64^c^ (131.54)	−40.33	−24.95	−38.36^a^	−38.36^a^	−21.99^a^	−21.99^a^	−34.30^c^
Relative humidity	−0.62^c^ (82.93)	−0.81	−0.42	1.22^ns^	1.22^ns^	–	–	–
Distance from street	−0.02^c^ (67.22)	−0.031	−0.01	–	–	–	–	–
–	–	–	–	R^2^ = 0.85 Adjust R^2^ = 0.75 RSME = 9.93	R^2^ = 0.85 Adjust R^2^ = 0.75 RSME = 9.93	R^2^ = 0.51 Adjust R^2^ = 0.43 RSME = 15.06	R^2^ = 0.51 Adjust R^2^ = 0.43 RSME = 15.06	R^2^ = 0.91 Adjust R^2^ = 0.89 RSME = 16.13

PM_10_	Simple Reg				Stepwise Reg			
	Coeff (Cons)		CI (95 %)		Forward stepwise reg (0.20)	Forward stepwise reg (0.10)	Backward stepwise reg (0.20)	Backward stepwise reg (0.10)
Height (0–500m)	−0.007^ns^ (106.04)		−0.06	0.05	–	–	–	–
Temperature	3.75^c^ (−38.66)		2.19	5.32	5.48^c^	3.75^c^	5.48^c^	3.75^c^
Relative humidity	−1.38^a^ (72.29)		−2.68	−0.08	1.10^ns^	–	1.10^ns^	–
Distance from street	−1.13^c^ (136.4)		−1.45	−0.81	–	–	–	–
–	–		–	–	R^2^ = 0.77 Adjust R^2^ = 0.73 RSME = 1.61	R^2^ = 0.71 Adjust R^2^ = 0.69 RSME = 1.74	R^2^ = 0.77 Adjust R^2^ = 0.73 RSME = 1.61	R^2^ = 0.71 Adjust R^2^ = 0.69 RSME = 1.74

NO_2_	Simple Reg				Stepwise Reg			
	Coeff (Cons)		CI (95 %)		Forward stepwise reg (0.20)	Forward stepwise reg (0.10)	Backward stepwise reg (0.20)	Backward stepwise reg (0.10)
Height (0–500m)	0.13^c^ (55.58)		0.08	0.19	–	–	–	–
Temperature	5.563^ns^ (119.77)		−18.92	30.05	−17.23^a^	−17.23^a^	–	–
Windspeed	−1.684^ns^ (35.13)		−7.89	4.52	−19.14^b^	−19.14^b^	–	–
Relative humidity	2.32^c^ (−21.03)		1.59	3.06	–	–	2.32^c^	2.32^c^
Distance from street	−0.01^c^ (63.77)		−0.02	−0.01	–	–	–	–
–	–		–	–	R^2^ = 0.91 Adjust R^2^ = 0.88 RSME = 14.3	R^2^ = 0.91 Adjust R^2^ = 0.88 RSME = 14.3	R^2^ = 0.86 Adjust R^2^ = 0.85 RSME = 16.22	R^2^ = 0.86 Adjust R^2^ = 0.85 RSME = 16.22

NO_**X**_	Simple Reg			Stepwise Reg				MEP#
	Coeff (Cons)	CI (95 %)		Forward stepwise reg (0.20)	Forward stepwise reg (0.10)	Backward stepwise reg (0.20)	Backward stepwise reg (0.10)	Forward stepwise reg (0.20)
Height (0–500m)	−0.11^c^ (71.25)	−0.14	−0.08	–	–	–	–	–
Temperature	−7.32^ns^ (274.92)	−24.45	9.80	–	–	–	–	–
Windspeed	−25.21^ns^ (174.47)	−96.49	46.05	–	–	–	–	−43.39^ns^
Relative humidity	2.14^ns^ (26.7)	−1.16	5.45	2.10^ns^	2.10^ns^	2.10^ns^	2.10^ns^	2.88^ns^
Distance from street	−0.01^ns^ (75.76)	−0.03	0.002	–	–	–	–	–
–	–	–	–	R^2^ = 0.77 Adjust R^2^ = 0.68 RSME = 27.70	R2 = 0.77 Adjust R^2^ = 0.68 RSME = 27.70	R^2^ = 0.77 Adjust R^2^ = 0.68 RSME = 27.70	R^2^ = 0.77 Adjust R^2^ = 0.68 RSME = 27.70	R^2^ = 0.58 Adjust R^2^ = 0.42 RSME = 37.57

ns: P > 0.05.

MER#: Meteorological parameters (height, temperature, windspeed, and relative humidity).

a P ≤ 0.05.

b P ≤ 0.01.

c P ≤ 0.001.

The primary objective of this study was to examine the variation of pollutant concentration with altitude. It is important to note that a comprehensive investigation is required on the connection between meteorological parameters and pollutant concentration, and we can presently report only one preliminary finding based on a limited number of studies.

There was a significant relationship between the distance from the street and decreased pollutant (PM_2.5_, PM_10_, and NO_2_) concentration. Also, there is a significant relationship between pollutant concentration and meteorological parameters. When the pollution source remains relatively constant, weather conditions become the dominant factor for the diffusion, transportation, and clearance of pollutants [10].

Temperature and relative humidity reduce PM_2.5_'s concentration, but wind speed has the most significant impact. Furthermore, a comprehensive understanding of meteorological parameters makes it possible to accurately estimate pollutant concentrations (PM_2.5_, PM_10_, and NO_2_) (R^2^ > 0.7).

Table 3 indicates the regression between air pollutant concentrations and specific heights. No relationship was found between the specified altitudes and air pollutants. At a height of 10–30 m, PM_2.5_ concentration increased with height, and at 30–60 m, PM_10_ concentrations decreased as height increased. The PM_2.5_/PM_10_ ratio and NO_2_ concentration increased as the height increased, but the NO_X_ concentration decreased.

**Table 3:** Regression of air pollutants with height (coefficients and Constant number (in parentheses)).

Altitude (m)	PM_2.5_	PM_10_	PM_2.5_/PM_10_	NO_2_	NO_X_
≤10	−2.20^ns^ (166.07)	−5.61^ns^ (170.46)	0.003^ns^ (0.66)	0.99^ns^ (52.86)	–
10–30	4.07^a^ (25.94)	1.91^ns^ (96.85)	0.01^ns^ (0.32)	−0.34^ns^ (69.57)	–
30–60	2.4^ns^ (13.91)	−0.87^a^ (96.73)	−0.0008^ns^ (0.72)	−0.12^ns^ (61.02)	–
60–100	0.85^ns^ (72.41)	−0.15^ns^ (68.58)	0.0004^ns^ (0.61)	−0.06^ns^ (57.52)	–
100–300	−0.06^a^ (79.16)	−0.01^ns^ (129.41)	0.0001^ns^ (0.67)	0.09^ns^ (41.74)	−0.23^b^ (89.38)
300–500	0.02^ns^ (40.09)	−0.002^ns^ (115.14)	0.0003^ns^ (0.68)	0.46^ns^ (−39.16)	–
0–500	−0.11^b^ (94.32)	−0.01^ns^ (107.29)	0.0008^b^ (0.49)	0.13^b^ (55.58)	−0.11^b^ (71.17)

ns: P > 0.05.

∗∗P ≤ 0.01.

a P ≤ 0.05.

b P ≤ 0.001.

Table 4 describes the concentration of pollutants at specific altitudes (by considering an increase of 1 m in height and dividing the difference in pollutant concentration by the base concentration, the concentrations of the following pollutants are computed). Pollutant concentration variations are more pronounced at lower altitudes.

**Table 4:** The concentration of pollutants at specific altitudes.

	Height	Min	Max	Mean	Sd	Median	Percentile [5]	Percentile (95)
- > PM_2.5_	≤10	−1.09	0.08	−0.54	0.47	−0.73	−1.06	0.04
	10–30	−1.83	1.74	−0.28	0.61	−0.13	−1.21	0.31
	30–60	−0.71	1.38	−0.11	0.41	−0.14	−0.61	0.66
	60–100	−0.53	0.66	−0.12	0.19	−0.14	−0.29	0.04
	100–300	−0.29	0.25	−0.08	0.09	−0.09	−0.18	0.04
	300–500	−0.18	0.03	−0.06	0.04	−0.06	−0.13	0.02
	Total	−1.83	1.74	−0.13	0.33	−0.09	−0.72	0.18
PM_10_	≤10	−4.86	−0.06	−1.71	1.76	−0.95	−4.49	−0.12
	10–30	−4.23	1.99	−1.00	1.31	−0.85	−3.20	0.61
	30–60	−0.88	0.52	−0.32	0.39	−0.44	−0.87	0.40
	60–100	−0.54	−0.04	−0.17	0.13	−0.15	−0.39	−0.04
	100–300	−0.29	0.00	−0.13	0.11	−0.06	−0.27	−0.01
	300–500	−0.15	0.25	0.01	0.13	−0.02	−0.12	0.18
	Total	−4.86	1.99	−0.59	1.06	−0.25	−3.12	0.18
NO_2_	0–30	−1.39	3.15	0.33	1.75	−0.19	−1.26	3.00
	30–60	−1.31	1.21	0.02	0.66	0.15	−1.06	0.85
	60–100	−0.90	0.67	0.02	0.39	−0.01	−0.54	0.59
	100–300	−0.26	0.30	−0.04	0.15	−0.05	−0.25	0.24
	300–500	−0.17	0.35	0.03	0.22	−0.09	−0.16	0.34
	Total	−1.39	3.15	0.04	0.68	−0.03	−0.98	0.65
NO_**X**_	100–300	−0.30	−0.12	−0.23	0.07	−0.24	−0.30	−0.14
	Total	−1.02	−0.12	−0.32	0.29	−0.24	−0.77	−0.13

## 3 Discussion

### 3.1 Vertical distribution of PM_2.5_ and PM_10_

In the present review of 42 studies that investigate vertical distribution of PM_2.5,_ 25 studies showed a decrease in the concentration with increasing the height. This generally happens in the atmosphere due to the dilution effect, which disperses the pollutants and reduces their concentration. However, this is not always the case, as some studies observed the contradictory trend since the pollutant can be transported vertically by atmospheric processes, leading to higher concentrations at higher altitudes. Additionally, local sources of pollution, such as factories or traffic, can create localized hotspots of higher concentration regardless of height [21]. In 9 studies, no fixed trend was observed, and several patterns were reported. The patterns included decrease to a certain altitude then increase, decreasing trend in a specific street, however no fixed pattern in another, growth to a certain height then decrease, rapid decline followed by gradual decline pattern, various patterns in different seasons, multiple trends during the day and at night, distinct patterns in clean and polluted days or clean and polluted area, different pattern in an industrial and residential area, and various trend in other of the day [4,16]. The mechanisms of PM_2.5_ formation at higher altitudes may differ from those at lower altitudes. Photochemical reactions are the primary reason controlling the variation of PM_2.5_ at higher altitudes. In comparison, the night chemistry and lower mixing layer heights are possibly the dominant reason controlling PM_2.5_ at lower altitudes. Rapid decrease with increasing height usually under conditions with high visibility, and slight decrease with increasing height generally during periods of severe pollution when the boundary layer usually had a temperature inversion and confined the pollutants within the boundary layer [21,50,70].

Therefore, the effect of height on pollutant concentration is complex and depends on multiple factors such as meteorological parameters, location measurement, and height of the planetary boundary layer (PBL). The PBL is the lowest part of the Earth's atmosphere, which is directly influenced by the Earth's surface. It is called "planetary" because the planet's rotation affects this layer's dynamics [9]. Pollutants from various sources mix in the lower atmosphere, where their vertical distribution changes throughout the day, creating a layer known as the mixing layer. Research indicates that the stability of the planetary boundary layer (PBL) can hinder pollutant mixing, influenced by wind speed and solar radiation. Higher air pollution episodes have been observed during temperature inversions across different geographical areas. These inversions impede convective air movement, restricting pollutant dispersion and trapping them within a limited air mass [9,71]. The PBL is characterized by turbulent air mixing due to heat, moisture, and momentum exchange between the surface and the atmosphere. This layer is responsible for transporting air pollutants and the dispersion of airborne particles and gases. The height of the PBL varies throughout the day and is influenced by the strength of solar radiation, surface characteristics (e.g., vegetation, urbanization), and atmospheric stability [9,71]. Most in the afternoon, the atmospheric stability decreases with the expansion of the PBL, resulting in increased vertical mixing of air pollutants. These findings suggest that the positive altitude dependence of PM_2.5_ concentrations in the middle or upper part of the PBL was likely due to transport rather than local emissions. PM_2.5_ concentrations at higher altitudes often peak in the afternoon due to the delay in vertical transport from the surface, which is driven by the expansion of the PBL [9,26]. During nighttime and early morning, the height of the PBL is typically low, which limits the upward transport of surface-level PM_2.5_ [51].

Occasionally, there is an upward trend in the concentration of particulate matter with increasing height (mainly in the winter). It happens more often when the atmosphere is stable and the boundary layer height is low; it facilitates the formation of a temperature inversion layer near the ground, impeding the upward dispersion of PM_2.5_. The thermal inversion layer is responsible for the inversion of PM_2.5_ concentrations at an altitude of around 300–400 m [51]. Under conditions of a stable atmosphere and a lower boundary layer height, the concentrations of PM_2.5_ increased as altitude increased [66]. As a known fact, a stable atmospheric condition in winter holds pollutants suspended for a longer duration in the atmosphere [34]. The increase in PM_2.5_ concentrations above 400 m in height can be attributed to long-distance transportation. The occasional rise in PM_2.5_ concentrations with altitude is primarily influenced by the presence of a temperature inversion layer or the effect of long-distance transportation [66]. PM_2.5_ concentrations increased with altitude, particularly in urban areas heavily impacted by traffic-related pollution. This phenomenon can be attributed to the photochemical formation of NO_2_ during the daytime, which promotes the oxidation of volatile organic compounds (VOCs) and leads to the formation of secondary particulate matter [31]. Secondary particulate matter is mainly produced by coagulating gas molecules and heterogeneous chemical reactions [32]. Secondary PM_2.5_ presented a generally increasing trend with the increase in height [37]. The concentration of PM_2.5_ at higher heights can float more quickly in the air than PM_2.5_ on the ground. The concentration on the ground will eventually decrease due to the sedimentation effect [38].

Moreover, in urban areas, buildings and other obstructions can disrupt the wind flow, hindering the dispersion of pollutants and preventing their dilution [33]. So, street geometry is a factor that increases the concentration of particles with height [34]. Also, it has been pointed out that as the aspect ratio (ratio of the height of buildings to the width of the street) increases (as the cavity becomes deeper), the outer stream of fresh air above the cavity becomes less efficient in moving pollutants [29]. Also, on the streets, the prevention of clean air from higher altitudes, which contains fewer particles, from mixing with polluted air from traffic at lower elevations is due to tree leaves and other barriers. Consequently, the concentration of particulate matter at lower altitudes of these barriers is higher than that of particles at altitudes near the surface [41].

Similar to PM_2.5_, in most studies, although the concentration of PM_10_ decreased with increasing altitude, no significant relationship was found between PM_10_ concentration and altitude. Coarse particles (PM_10_) are primarily produced through physical processes such as grinding and crushing and can be removed more quickly through settling due to gravity [28]. However, we found that the PM_2.5_/PM_10_ ratio increased as the altitude increased, while the concentration of PM_2.5_ decreased with increasing altitude. This indicates that the decrease in concentration of PM_10_ is more pronounced than that of PM_2.5_.

The vertical distribution of fine particles was more uniform than coarse particles. The rate of concentration variation was more significant at lower altitudes than at higher altitudes. The reduction rate of particulate matter decreased gradually and reached a more consistent level after reaching 100 m [32].

Urban planners must account for the vertical distribution of pollutants when assigning residential zones. Areas at lower altitudes with elevated PM_2.5_ levels may need more stringent air quality regulations or could be reserved for non-residential purposes. Also, expanding green spaces and vegetation can reduce PM_2.5_ levels through natural filtration. Urban forests and green roofs are especially effective in lowering particulate matter [35,72]. Due to higher particulate matter concentrations at lower elevations, residents in these areas should consider using air purifiers if their exposure exceeds standard levels.

### 3.2 Vertical distribution of NO_2_ and NO_X_

In this study, it was observed that the concentration of NO_2_ increased with increasing elevation, whereas the concentration of NO_**X**_ decreased. The concentrations of NO_2_ exhibited a height-dependent rise, particularly during periods of stable atmospheric conditions and a low boundary layer height. This facilitated the formation of a temperature inversion layer near the surface. The increase in NO_2_ concentrations, such as PM_2.5_ above 400 m in height, can be attributed to long-distance transportation [66]. Also, when ozone descends from the upper layers of the atmosphere, it can react with NO_**X**_ to produce NO_2_ At high altitudes [44,54]. NO_**X**_ released from the surface is mainly confined to a thin layer close to the ground [36].

Occasionally, the concentration of NO2 at low altitudes (less than 50 m) with increasing elevation increased initially, followed by a decreasing trend. This indicates that vehicles' contribution to NO_2_ emissions is significant due to their lower emission height [12]. The complex profiles of NO_2_ concentrations are likely due to the mixing of NO-rich traffic emissions from ground level with ozone-rich air from higher altitudes. This mixing results in the formation of NO_2_ at intermediate heights [53].

Ensuring proper ventilation systems that filter out PM and NO_2_ for high-rise buildings can protect residents from exposure. Building designs that promote natural ventilation can also help reduce indoor pollutant levels [43,73].

Enforcing more rigorous emission standards for vehicles and industries, particularly in higher-altitude urban regions, can lower NO_2_ levels. Promoting the adoption of electric cars and public transportation can also be advantageous. Continuous air quality monitoring at different altitudes can help identify pollution hotspots and enforce regulations more effectively. Additionally, residents at higher altitudes should consider using air purifiers to minimize NO_2_ exposure if necessary.

### 3.3 Strength and limitation

To our knowledge, this is the first time a comprehensive review of studies on the vertical distribution of air pollutants has been done. Because the observations reported in the studies were very variable, one of the strengths is that performing statistical analysis made the result more reliable. Furthermore, the inclusion of meteorological parameters and also the distance from the street allowed for a comprehensive understanding of how these factors interact with altitude in affecting pollutant dispersion, leading to a more accurate conclusion regarding the relationship between pollutant concentration and altitude. Although we presented preliminary findings in this field based on a limited number of studies, this study showed that comprehensive research on the issue of the relationship between meteorological parameters, altitude, and pollutant concentration is needed.

We removed articles not written in English and excluded those for which full-text access was unavailable. We derived most of the data from the graph; precise numbers could yield more accurate results.

## 4 Conclusion

This study conducted a systematic review to investigate the vertical distribution of concentration for air pollutants (PM_2.5_, PM_10_, NO_2_, and NO_**X**_) up to 500 m high. As the altitude increased, PM_2.5_ and NO_**X**_ concentrations declined, whereas NO_2_ concentrations increased. There was no significant relationship between height and PM_10_ concentration. However, the ascending trend in the PM_2.5_/PM_10_ ratio with increasing altitude indicates a steeper decline in PM_10_ concentrations compared to PM_2.5_ concentrations. Under specific circumstances, there is an upward trend in the concentration of particulate matter with increasing altitude. This can be attributed to factors such as secondary particulate matter formation, temperature inversion, long-distance transport of particulate matter, the height of the PBL, and the presence of buildings and obstructions in urban areas that disrupt wind flow. The chemical reactions between ozone and NO_**X**_ produce NO_2_, which is a primary factor contributing to the rise in carbon concentrations with increasing altitude. Creating thorough air quality management plans that account for the vertical distribution of pollutants can result in more effective strategies to minimize human exposure. This involves establishing altitude-specific air quality standards and guidelines.

Future studies can examine the vertical distribution of ozone concentration and explore the impact of meteorological parameters on pollutant concentrations. Encouraging further research into the vertical distribution of air pollutants and their health impacts can provide valuable data for refining air quality management practices. Innovations in pollution control technologies and urban design can also contribute to healthier urban environments.

## CRediT authorship contribution statement

**Vahid Roostaei:** Writing – review & editing, Writing – original draft, Investigation, Formal analysis, Conceptualization. **Farzaneh Gharibzadeh:** Writing – review & editing, Writing – original draft, Investigation, Conceptualization. **Mansour Shamsipour:** Writing – review & editing, Formal analysis. **Sasan Faridi:** Writing – review & editing. **Mohammad Sadegh Hassanvand:** Writing – review & editing, Writing – original draft, Investigation, Formal analysis, Conceptualization.

## Data and code availability statement

Data included in article/supplementary material is referenced in the article.

## Additional files

Raw data and supplementary data are available.

## Other information

The review was not registered and protocol was not prepared.

## Funding

Center for Air Pollution Research, Institute for Environmental Research, Tehran University of Medical Sciences.

## Declaration of competing interest

The authors declare that they have no known competing financial interests or personal relationships that could have appeared to influence the work reported in this paper.
