# Supplementary material for: Vertical distribution of ambient air pollutants (PM2.5, PM10, NOX, and NO2); A systematic review
Source: Heliyon. 2024 Oct 24;10(21):e39726. doi: 10.1016/j.heliyon.2024.e39726 (PMC11564963; doi:10.1016/j.heliyon.2024.e39726)
Supplement: Multimedia component 1 [file mmc1.docx]

**Vertical distribution of ambient air pollutants (PM_2.5_, PM_10_, NO_X_, and NO_2_); A systematic review**

Vahid Roostaei^1^, Farzaneh Gharibzadeh^1^, Mansour Shamsipour^3^, Sasan Faridi^1,^ ^2^, Mohammad Sadegh Hassanvand^1, 2^*

^1^ Department of Environmental Health Engineering, School of Public Health, Tehran University of Medical Sciences, Tehran, Iran

^2^ Center for Air Pollution Research (CAPR), Institute for Environmental Research (IER), Tehran University of Medical Sciences, Tehran, Iran

^3^Department of Research Methodology and Data Analysis, Institute for Environmental Research (IER), Tehran University of Medical Sciences, Tehran, Iran

**^⁎^** **Corresponding Author:** MS. Hassanvand, Ph.D., Center for Air Pollution Research (CAPR), Institute for Environmental Research (IER) and Department of Environmental Health Engineering, School of Public Health, Tehran University of Medical Sciences, Tehran, Iran ([hassanvand@tums.ac.ir](mailto:Hassanvand@tums.ac.ir)).

Table 1. Country, city, number, and year of studies for the vertical distribution of PM, NO_2,_ and NO**_X_**

| NO. | Country/region | City/Region | Number of studies | Year |
| --- | --- | --- | --- | --- |
| 1 | China | Shandong  Beijing  Hong Kong  Guangzhou  Shanghai  Changchun  Xi’an  Tianjin  Luoyang  Lanzhou  Pearl River Delta  Yangtze River Delta  Macao  Nanjing  Lin’an  Longfengshan  Raoyang  Wangdu  Fengxian district  Lushan mountain  Coast of Bohai Sea | 1  5  1  3  7  1  2  3  1  1  2  2  2  1  1  1  1  1  1  1  1 | 2020  2005, 2005, 2006, 2008, 2021  2000  2003, 2015, 2020  2007, 2017, 2018, 2020, 2021, 2021, 2021  2021  2018, 2021  2009, 2011, 2015  2021  2019  2020, 2022  2018, 2022  2002, 2020  2018  2019  2023  2022  2021  2021  2021  2022 |
| 2 | Greece | Patras | 1 | 2020 |
| 3 | India | Chennai  Delhi | 2  1 | 2015, 2021  2021 |
| 4 | Philippines | Quezon City | 1 | 2018 |
| 5 | Japan | Tokyo | 2 | 2011, 2014 |
| 6 | Italy | Bologna | 1 | 2018 |
| 7 | USA | Texas | 1 | 2004 |
| 8 | Thailand | Bangkok | 1 | 2020 |
| 9 | Singapore | Singapore | 2 | 2007, 2009 |
| 10 | Taiwan | Taichung  Taipei | 2  4 | 2002, 2020  2018, 2020, 2021, 2023 |

Table S2. Summary of the results of each study

| **Study** | **Height (m)*** | **Measurement Time** | **Pollutant** | **Range of pollutants (µg/m^3^)** | **Summary of study result** |
| --- | --- | --- | --- | --- | --- |
| (1) | 92,158,195,236,273,312,352,394,441 | Day | PM_2.5_ | 4.17-7.68 | Decrease to 195m, then increase to 273m, then decrease |
| (2) | 0,40,60,120,240,360,500 | Day | PM_2.5,_ PM_10_ | PM_2.5_: 2.2-9.6  PM_10_: 5.5-19.5 | Decrease and remain stable after 120 m |
| (3) | 8,100,200,325 | Both | PM_2.5,_ PM_10_ | PM_2.5_: 75-106.5  PM_10_: 139.4-169.1 | Decrease |
| (4) | In canyon street (2.5,14,21,26)  In open streets (2.5,8,10,18,25) | Both | PM_2.5_  PM_10_ | PM_2.5_: 68.4-129.2  PM_10_: 63.6-166.8 | In Street Canyon, there was decreased  In open streets, there is no fixed pattern |
| (5) | 1,13,100,300,500 | Both | NO_2_ | 39.4-73.3 | Increased to 50 m, then decreased |
| (6) | 6,16,27 | Both | PM_2.5,_ PM_10_ | PM_2.5_: 18-48  PM_10_: 24-76 | In winter 16˃6˃27  In summer 6˃16˃27 |
| (7) | 30,75,110 | Both | PM_2.5_ | 2.9-10.07 | In day: increase  At night 75˃30˃110 |
| (8) | 121,454 | NR | PM_2.5,_ PM_10,_ | PM_2.5_: 19.2-48.9  PM_10_: 23.4-55.6 | decreased |
| (9) | 8,100,200, 320 | NR | PM_2.5,_ PM_10_  NO_2_ | PM_2.5_: 3.5-11  PM_10_: 17.7-169.8  NO_2_: 18-60 | In calm weather or on polluted days, it gradually decreases.  On clean days or weak cold days: rapidly decreasing |
| (10) | 14.5,28.4,42.3,56.2 | Both | PM_2.5,_ PM_10_ | PM_2.5_: 18-85  PM_10_: 45-127 | PM_2.5_: 14.5 m-42.3 m increased then decreased  PM_10_: decreased |
| (11) | 3,16,17,37,38 | NR | PM_2.5,_ PM_10_ | PM_2.5_: 9-69  PM_10_: 20-185 | Winter, PM_2.5_ increased with increasing height, PM_10_ increased then decreased at 38m  In summer, Both PM_2.5_ and PM_10_ decrease with increasing height. |
| (12) | 9,15,21,24,27,33,39,45 | Both | PM_2.5_ | 54.5-87.5 | decreased |
| (13) | 0,50,100,150, …., 500 | NR | PM_2.5,_ PM_10,_ NO_2_ | PM_2.5_: 37-198  PM_10_: 50-200  NO_2_: 141-648.6 | PM_2.5_ and PM_10_ increased in industrial areas; they decreased and then increased near small-scale industrial and residential places. NO2 increased |
| (14) | 0-100 | Day | PM_2.5_ | 189-500 | At 8 A.M., decreased sharply at other hours decreased gradually |
| (15) | 2-40-120-220 | Both | NO**_X_** | 13.16-122.2 | decreased |
| (16) | 2-40-120-220 | Both | PM_2.5,_ PM_10_ | PM_2.5_: 59-112  PM_10_: 80-140 | PM_10_: decreases  PM_2.5_: 10 A.M., 6 P.M. increased  Other time decreased |
| (17) | 40-220-390 | Both | PM_2.5_ | 4-38 | 220˃40˃390 |
| (18) | 50, 100, 150, …, 500 | Day | NO_2_ | 0.7-4.5 | decreased |
| (19) | 3,12,24,36,48,60,72,84,  96 | Both | NO_2,_ PM_2.5_ | PM_2.5_: 51.5-88  NO_2_: 38-70.5 | NO_2_: decreased for windows facing traffic but insignificant for windows away from traffic.  PM_2.5:_ decreased for windows facing away from traffic but increased for windows facing traffic in fall |
| (20) | 12,30,42,51 | Both | PM_2.5_ | 26.97-75.91 | 30˃12˃42  30˃12˃51 |
| (21) | 60,460,280 | Day | NO_2_ | 16.54-61.66 | decreased |
| (22) | 3,16,21,28 | Both | PM_10_ | 36.9-81.93 | 3˃28˃21 ˃16 |
| (23) | 24,220 | Both | PM_2.5_ | 11-22.7 | decreased |
| (24) | 70,120,220,335 | Both | PM_2.5,_ NO**_X_** | PM_2.5_: 21.5-149  NO_2_: 28-194 | decreased |
| (25) | 1,50,100, …, 500 | Day | PM_2.5_ | 18.5-109 | 8 (hour),9,10 increased  11, 15, 16, 17 decreased |
| (26) | 0,50,100, …, 500 | Day | PM_2.5,_ NO_2_ | PM_2.5_: 25-120  NO_2_: 2.06-15.04 | At 8:10, 10:10,15:20 decreased  At 16:50 decreased and then increased |
| (27) | 1,300,400,500 | Day | PM_2.5_ | 11-177 | decreased |
| (28) | 1.5,8,20,38 | Day | PM_2.5_ | 122-375 | 30_31 May (high wind speed and step-up canyon) no different  23_25 November (low wind speed and wind blowing parallel to the canyon) decreased |
| (29) | 1.5_8, 17_20, 29_32 | Both | PM_2.5_ | 15-16 | 1.5_8˃29_32˃17_20 |
| (30) | 1.5_8, 17_20, 29_32 | Both | PM_2.5_ | 12.10-12.30 | decreased |
| (31) | 0_500 | Night | PM_2.5_ | 19.5-46-5 | decreased |
| (32) | 1.5,10,25,35,50,65,80,100 | Day | PM_2.5,_ PM_10_ | PM_2.5_: 24.7-80  PM_10_: 35-124.5 | forest park decreased  downtown decreased, but in streets with tall trees, peak value was observed at 25 m |
| (33) | 1.5,3,5,7,9,11,13,15,17 | Day | PM_10_ | 111-152 | Decreased  In the street with vegetation and  noise barriers maximum at 7m  in streets with vegetation, noise barriers, and buildings and streets without vegetation, noise  barriers and buildings rise slightly, then decreased |
| (34) | 0,300,400,500 | Day | PM_2.5_ | 17-135 | decreased |
| (35) | 8,15,47,80,100,140,200,240,320 | Both | NO_2_ | 10-111 | Three groups  1st (11 to 16 January): increasing trend with increasing height, and reached a maximum at 47 m, remaining higher concentrations at 80, 100, and 140 m, followed by a rapid decrease above 200 m with a daily minimum at 320 m  2nd (21 February to 2 March): decreased at 15 m, then concentrations increased with height, took highest values at 80 m, and decreased upwards  3rd (19 to 29 March): increased gradually from the ground to 15 m and 47 m, and a decreasing trend extended to an upper level of 325 m |
| (36) | 0.25,0.5,1,1.5,2 | Day | NO_2_ | 45.12-70.5 | No significant height dependence was observed |
| (37) | 5_10,30_45 | Both | PM_2.5,_ PM_10_ | PM_2.5_: 79.32-120.9  PM_10_: 135.1-258.6 | decreased |
| (38) | 2,15,26,44,66 | Both | PM_2.5,_ NO_2_ | PM_2.5_: 14-27  NO_2_: 18.1-70.5 | decreased |
| (39) | 2,21,57,85,107 | Night | NO_2_ | 9.30-35.72 | Maximum near ground |
| (40) | 0, 100, 200, 300, 400, 500 | Day | PM_2.5_ | 60.3-92 | decreased |
| (41) | 2,8,19,30,59,79 | Day | PM_2.5,_ PM_10_ | PM_2.5_: 70-205  PM_10_: 60-620 | decreased |
| (42) | 5,15,25,37 | Both | NO_2_ | 16-139 | decreased |
| (43) | 1.5,23.1,36.3,56.1,75.9,89.1 | Both | PM_2.5,_ PM_10_ | PM_2.5_: 14.8-35  PM_10_: 17-57 | At 8:00 PM_10_ increased then decreased.  PM_2.5_ decreased and then increased  At 12:00, it first increased, then decreased, then increased again.  At 15:00 PM_10_ decreased, then increased, then decreased again.  PM_2.5_ decreased and then increased  At 18:00 PM_10_ decreased, then increased, then decreased again.  PM_2.5_ decreased  At 22:00 PM_10_ increased then decreased.  PM_2.5_ decreased then increased. |
| (44) | 10,40,120,220 | Both | PM_10_ | 80-140 | decreased |
| (45) | 0,5,10,15,20,30,40,50,60,70,80,90, 100,110,120 | Both | PM_2.5,_ PM_10_ | PM_2.5_: 10.5-30  PM_10_: 18-42.6 | decreased |
| (46) | 0,118,168,488 | Both | PM_2.5_ | 11-105 | Decreased |
| (47) | 14,32,53 | Day | PM_2.5_ | 46525- 195280  (counts/liter) | 32˃14˃53 |
| (48) | 8,120,28 | Both | NO**_X_**, NO_2_ | NO**_X_**:2-180  NO_2_: 3-91 | decreased |
| (49) | 0, 5.5, 6.5, 11.5, 12.5, 16.5, 17.5, 34.5 | Day | PM_2.5_ | 0.66-0.75 | decreased |
| (50) | 300-400-500 | Both | PM_2.5_ | 27-36 | decreased |
| (51) | 0, 20, 40, …, 500 | Day | PM_2.5_ | 10-190 | decreased |
| (52) | 10-500 | Day | PM_2.5_ | 25-150 | Three vertical profiles: A (convective state): PM_2.5_ decreased, B (stable state): PM_2.5_ sharply decreased, C (multilayer structure): Certain pollutants remained suspended in the higher atmospheric layer. |
| (53) | 0, 50, 100, …, 500 | Day | PM_2.5_, NO_2_ | PM_2.5:_ 2-27  NO_2_: 3.76-28.2 | Generally decreased, but NO_2_ and PM_2.5_ sometimes increased with heigh |
| (54) | 30, 200, 400 | Day | NO_2_ | 3.27-14.34 | decreased |
| (55) | 0, 100, …, 500 | Day | NO_2_ | 7.52-28.2 | Increased to 100 m, then decreased |
| (56) | 1.5, 20, 31, 42, 52, 63 | NR | PM_2.5_ | 7.2-7.9 | decreased |
| (57) | 0, 200, 400 | Day | NO_2_ | 1.3-2.3 | decreased |

*Altitudes greater than 500 m are not considered.

Figure S1. Temporal distribution of included studies (n= 57) according to publication year

Figure S2. Measurement heights of articles

**References**

1. Babaan JB, Ballori JP, Tamondong AM, Ramos RV, Ostrea PM, editors. Estimation of PM 2.5 vertical distribution using customized UAV and mobile sensors in Brgy. UP Campus, Diliman, Quezon City. International Archives of the Photogrammetry, Remote Sensing and Spatial Information Sciences - ISPRS Archives; 2018.

2. Cao R, Li B, Wang HW, Tao S, Peng ZR, He HD. Vertical and horizontal profiles of particulate matter and black carbon near elevated highways based on unmanned aerial vehicle monitoring. Sustainability (Switzerland). 2020;12(3).

3. Chan CY, Xu XD, Li YS, Wong KH, Ding GA, Chan LY, et al. Characteristics of vertical profiles and sources of PM2.5, PM10 and carbonaceous species in Beijing. Atmospheric Environment. 2005;39(28):5113-24.

4. Chan LY, Kwok WS. Vertical dispersion of suspended particulates in urban area of Hong Kong. Atmospheric Environment. 2000;34(26):4403-12.

5. Chen CL, Tsuang BJ, Tu CY, Cheng WL, Lin MD. Wintertime vertical profiles of air pollutants over a suburban area in central Taiwan. Atmospheric Environment. 2002;36(12):2049-59.

6. Chen HL, Li CP, Tang CS, Lung SCC, Chuang HC, Chou DW, et al. Risk assessment for people exposed to pm2.5 and constituents at different vertical heights in an urban area of taiwan. Atmosphere. 2020;11(11).

7. Choomanee P, Bualert S, Thongyen T, Salao S, Szymansk WW, Rungratanaubon T. Vertical Variation of Carbonaceous Aerosols within the PM2.5 Fraction in Bangkok, Thailand. Aerosol and Air Quality Research. 2020;20(1):43-52.

8. Deng X, Li F, Li Y, Li J, Huang H, Liu X. Vertical distribution characteristics of PM in the surface layer of Guangzhou. Particuology. 2015;20:3-9.

9. Ding G, Chan C, Gao Z, Yao W, Li Y, Cheng X, et al. Vertical structures of PM10 and PM2.5 and their dynamical character in low atmosphere in Beijing urban areas. Science in China, Series D: Earth Sciences. 2005;48(SUPPL.2):38-54.

10. Ezhil Kumar MR, Karthikeyan S. Study on vertical profiling of particulates in ambient air, exposure assessment of inhabitants in high rise buildings and assessment of AQI, in Chennai City. Indian Journal of Environmental Protection. 2015;35(5):395-406.

11. Ezhilkumar MR, Karthikeyan S, Aswini AR, Hegde P. Seasonal and vertical characteristics of particulate and elemental concentrations along diverse street canyons in South India. Environmental science and pollution research international. 2021.

12. Gao Y, Wang Z, Lu QC, Liu C, Peng ZR, Yu Y. Prediction of vertical PM2.5 concentrations alongside an elevated expressway by using the neural network hybrid model and generalized additive model. Frontiers of Earth Science. 2017;11(2):347-60.

13. Guan R, Yu J, Li M, Yan J, Liu Z. Preparation of electrochemical sensor assisted unmanned aerial vehicles system for SO2, O3, NO2, CO and PM2.5/PM10 detection in air. International Journal of Electrochemical Science. 2021;16:1-9.

14. Han L, Zhao J, Zhang J, Gao Y, Xin K. Vertical distribution of urban near-surface pollutant PM2.5 based on UAV monitoring platform. Chemical Engineering Transactions. 2018;71:25-30.

15. Han S, Bian H, Tie X, Xie Y, Sun M, Liu A. Impact of nocturnal planetary boundary layer on urban air pollutants: Measurements from a 250-m tower over Tianjin, China. Journal of Hazardous Materials. 2009;162(1):264-9.

16. Han S, Zhang Y, Wu J, Zhang X, Tian Y, Wang Y, et al. Evaluation of regional background particulate matter concentration based on vertical distribution characteristics. Atmospheric Chemistry and Physics. 2015;15(19):11165-77.

17. Ho WY, Tseng KH, Liou ML, Chan CC, Wang CH. Application of Positive Matrix Factorization in the Identification of the Sources of PM2.5 in Taipei City. International Journal of Environmental Research and Public Health. 2018;15(7).

18. Ji X, Hu Q, Hu B, Wang S, Liu H, Xing C, et al. Vertical structure of air pollutant transport flux as determined by ground-based remote sensing observations in fen-wei plain, china. Remote Sensing. 2021;13(18).

19. Jin L, Berman JD, Warren JL, Levy JI, Thurston G, Zhang Y, et al. A land use regression model of nitrogen dioxide and fine particulate matter in a complex urban core in Lanzhou, China. Environ Res. 2019;177:108597.

20. Kalaiarasan M, Balasubramanian R, Cheong KWD, Tham KW. Traffic-generated airborne particles in naturally ventilated multi-storey residential buildings of Singapore: Vertical distribution and potential health risks. Building and Environment. 2009;44(7):1493-500.

21. Kang Y, Tang G, Li Q, Liu B, Cao J, Hu Q, et al. Evaluation and Evolution of MAX-DOAS-observed Vertical NO2 Profiles in Urban Beijing. Advances in Atmospheric Sciences. 2021;38(7):1188-96.

22. Karkoulias VA, Marazioti PE, Georgiou DP, Maraziotis EA. Computational Fluid Dynamics modeling of the trace elements dispersion and comparison with measurements in a street canyon with balconies in the city of Patras, Greece. Atmospheric Environment. 2020;223.

23. Kudo S, Sekiguchi K, Kim KH, Sakamoto K. Spatial distributions of ultrafine particles and their behavior and chemical composition in relation to roadside sources. Atmospheric Environment. 2011;45(35):6403-13.

24. Li L, Lu C, Chan PW, Zhang X, Yang HL, Lan ZJ, et al. Tower observed vertical distribution of PM2.5, O3 and NOx in the Pearl River Delta. Atmospheric Environment. 2020;220.

25. Li XB, Peng ZR, Wang D, Li B, Huangfu Y, Fan G, et al. Vertical distributions of boundary-layer ozone and fine aerosol particles during the emission control period of the G20 summit in Shanghai, China. Atmospheric Pollution Research. 2021;12(1):352-64.

26. Li XB, Wang D, Lu QC, Peng ZR, Fu Q, Hu XM, et al. Three-dimensional analysis of ozone and PM2.5 distributions obtained by observations of tethered balloon and unmanned aerial vehicle in Shanghai, China. Stochastic Environmental Research and Risk Assessment. 2018;32(5):1189-203.

27. Li XB, Wang DS, Lu QC, Peng ZR, Wang ZY. Investigating vertical distribution patterns of lower tropospheric PM2.5 using unmanned aerial vehicle measurements. Atmospheric Environment. 2018;173:62-71.

28. Li XL, Wang JS, Tu XD, Liu W, Huang Z. Vertical variations of particle number concentration and size distribution in a street canyon in Shanghai, China. Science of the Total Environment. 2007;378(3):306-16.

29. Liao HT, Chang JC, Tsai TT, Tsai SW, Chou CCK, Wu CF. Vertical distribution of source apportioned PM(2.5)using particulate-bound elements and polycyclic aromatic hydrocarbons in an urban area. Journal of Exposure Science and Environmental Epidemiology. 2020;30(4):659-69.

30. Liao HT, Lee CL, Tsai WC, Yu JZ, Tsai SW, Chou CCK, et al. Source apportionment of urban PM2.5 using positive matrix factorization with vertically distributed measurements of trace elements and nonpolar organic compounds. Atmospheric Pollution Research. 2021;12(4):200-7.

31. Liu B, Wu C, Ma N, Chen Q, Li Y, Ye J, et al. Vertical profiling of fine particulate matter and black carbon by using unmanned aerial vehicle in Macau, China. Sci Total Environ. 2020;709:136109.

32. Liu F, Zheng X, Qian H. Comparison of particle concentration vertical profiles between downtown and urban forest park in Nanjing (China). Atmospheric Pollution Research. 2018;9(5):829-39.

33. Liu X, Shi XQ, He HD, Li XB, Peng ZR. Vertical distribution characteristics of particulate matter beside an elevated expressway by unmanned aerial vehicle measurements. Building and Environment. 2021;206.

34. Lu SJ, Wang DS, Wang ZY, Li B, Peng ZR, Li XB, et al. Investigating the Role of Meteorological Factors in the Vertical Variation in PM2.5 by Unmanned Aerial Vehicle Measurement. Aerosol and Air Quality Research. 2019;19(7):1493-507.

35. Meng ZY, Ding GA, Xu XB, Xu XD, Yu HQ, Wang SF. Vertical distributions of SO2 and NO2 in the lower atmosphere in Beijing urban areas, China. Science of the Total Environment. 2008;390(2-3):456-65.

36. Nakashima Y, Jones CE, Yamanobe W, Kajii Y. Near-surface vertical profiles of urban roadside NOx and fine particles. Aerosol and Air Quality Research. 2014;14(6):1763-8.

37. Priyan RS, Peter AE, Menon JS, George M, Nagendra SMS, Khare M. Vertical distribution of PM10 and PM2.5 emission sources and chemical composition during winter period in Delhi city. Air Quality Atmosphere and Health.

38. Sajani SZ, Marchesi S, Trentini A, Bacco D, Zigola C, Rovelli S, et al. Vertical variation of PM2.5 mass and chemical composition, particle size distribution, NO2, and BTEX at a high rise building. Environmental Pollution. 2018;235:339-49.

39. Stutz J, Alicke B, Ackermann R, Geyer A, White A, Williams E. Vertical profiles of NO3, N2O5, O3, and NOx in the nocturnal boundary layer: 1. Observations during the Texas Air Quality Study 2000. Journal of Geophysical Research D: Atmospheres. 2004;109(12):D12306 1-14.

40. Wang D, Wang Z, Peng ZR, Wang D. Using unmanned aerial vehicle to investigate the vertical distribution of fine particulate matter. International Journal of Environmental Science and Technology. 2020;17(1):219-30.

41. Wu Y, Hao JM, Fu LX, Wang ZS, Tang U. Vertical and horizontal profiles of airborne particulate matter near major roads in Macao, China. Atmospheric Environment. 2002;36(31):4907-18.

42. Xie S, Zhang Y, Qi L, Tang X. Spatial distribution of traffic-related pollutant concentrations in street canyons. Atmospheric Environment. 2003;37(23):3213-24.

43. Zhang X, Fan YS, Yu WQ, Wang H, Niu BB, Li MY. Vertical Distribution Characteristics of Outdoor Particles Concentrations in High-Rise Buildings. Polish Journal of Environmental Studies. 2021;30(2):1913-22.

44. Zhang YF, Xu H, Tian YZ, Shi GL, Zeng F, Wu JH, et al. The study on vertical variability of PM10 and the possible sources on a 220 m tower, in Tianjin, China. Atmospheric Environment. 2011;45(34):6133-40.

45. Zheng T, Li B, Li XB, Wang Z, Li SY, Peng ZR. Vertical and horizontal distributions of traffic-related pollutants beside an urban arterial road based on unmanned aerial vehicle observations. Building and Environment. 2021;187.

46. Zhou SZ, Wu LL, Guo JC, Chen WH, Wang XM, Zhao J, et al. Measurement report: Vertical distribution of atmospheric particulate matter within the urban boundary layer in southern China - size-segregated chemical composition and secondary formation through cloud processing and heterogeneous reactions. Atmospheric Chemistry and Physics. 2020;20(11):6435-53.

47. Cheong KWD, Balasubramanian R, Kalaiarasan M, editors. Field-based investigation on vertical distribution of airborne particulate matter in multi-storey buildings. IAQVEC 2007 Proceedings - 6th International Conference on Indoor Air Quality, Ventilation and Energy Conservation in Buildings: Sustainable Built Environment; 2007.

48. Sun Y, Wang YS, Li X, An JL, Xin JY, Hu B. An analysis for vertical distribution of O3, NOx and CO in the atmosphere during a serious air pollution in Beijing. Acta Geophysica Sinica. 2006;49(6):1616-22.

49. Wu CD, MacNaughton P, Melly S, Lane K, Adamkiewicz G, Durant JL, et al. Mapping the vertical distribution of population and particulate air pollution in a near-highway urban neighborhood: Implications for exposure assessment. Journal of Exposure Science and Environmental Epidemiology. 2014;24(3):297-304.

50. Duan J, Chen Y, Wang W, Li J, Zhang X, Lu G, et al. Cable-car measurements of vertical aerosol profiles impacted by mountain-valley breezes in Lushan Mountain, East China. Science of the Total Environment. 2021;768.

51. Song RF, Wang DS, Li XB, Li B, Peng ZR, He HD. Characterizing vertical distribution patterns of PM2.5 in low troposphere of Shanghai city, China: Implications from the perspective of unmanned aerial vehicle observations. Atmospheric Environment. 2021;265.

52. Sun H, Shi Y, Liu L, Ding W, Zhang Z, Hu F. Impacts of Atmospheric Boundary Layer Vertical Structure on Haze Pollution Observed by Tethered Balloon and Lidar. Journal of Meteorological Research. 2021;35(1):209-23.

53. Chen L, Pang X, Li J, Xing B, An T, Yuan K, et al. Vertical profiles of O3, NO2 and PM in a major fine chemical industry park in the Yangtze River Delta of China detected by a sensor package on an unmanned aerial vehicle. Science of the Total Environment. 2022;845.

54. Cheng S, Jin J, Ma J, Lv J, Liu S, Xu X. Temporal Variation of NO2 and HCHO Vertical Profiles Derived from MAX-DOAS Observation in Summer at a Rural Site of the North China Plain and Ozone Production in Relation to HCHO/NO2 Ratio. Atmosphere. 2022;13(6).

55. Xing C, Liu C, Hong Q, Liu H, Wu H, Lin J, et al. Vertical distributions and potential sources of wintertime atmospheric pollutants and the corresponding ozone production on the coast of Bohai Sea. Journal of Environmental Management. 2022;319.

56. Liao HT, Lai YC, Chao HJ, Wu CF. Vertical Characteristics of Potential PM2.5 Sources in the Urban Environment. Aerosol and Air Quality Research. 2023;23(3).

57. Liu S, Cheng S, Ma J, Xu X, Lv J, Jin J, et al. MAX-DOAS Measurements of Tropospheric NO2 and HCHO Vertical Profiles at the Longfengshan Regional Background Station in Northeastern China. Sensors. 2023;23(6).
